# Dr. McLeod

**Published:** 1861-01

**Authors:** 


DR. MCLEOD.—It affords us pleasure to see that Dr. McLeod, author of the Notes on the Surgery in the Crimea, elaborately noticed in this journal a year ago, has been appointed Professor of Surgery in the Andersonian University at Glasgow. A more able gentleman could not have been selected for this important trust.

